# Development and Internal Validation of a Disability Algorithm for Multiple Sclerosis in Administrative Data

**DOI:** 10.3389/fneur.2021.754144

**Published:** 2021-11-02

**Authors:** Ruth Ann Marrie, Qier Tan, Okechukwu Ekuma, James J. Marriott

**Affiliations:** ^1^Department of Internal Medicine, Max Rady College of Medicine, Rady Faculty of Health Sciences, University of Manitoba, Winnipeg, MB, Canada; ^2^Department of Community Health Sciences, Max Rady College of Medicine, Rady Faculty of Health Sciences, University of Manitoba, Winnipeg, MB, Canada; ^3^Manitoba Centre for Health Policy, Max Rady College of Medicine, Rady Faculty of Health Sciences, University of Manitoba, Winnipeg, MB, Canada

**Keywords:** multiple sclerosis, disability, administrative data, validation, health care utilization

## Abstract

**Objective:** We developed and internally validated an algorithm for disability status in multiple sclerosis (MS) using administrative data.

**Methods:** We linked administrative data from Manitoba, Canada to a clinical dataset with Expanded Disability Status Scale (EDSS) scores for people with MS. Clinical EDSS scores constituted the reference standard. We created candidate indicators using the administrative data. These included indicators based on use of particular health care services (home care, long-term care, rehabilitation admission), use of specific diagnostic codes (such as spasticity, quadriplegia), and codes based on use of Employment and Income Insurance. We developed algorithms to predict severe disability (EDSS ≥6.0), and to predict disability as a continuous measure. We manually developed algorithms, and also employed regression approaches. After we selected our preferred algorithms for disability, we tested their association with health care use due to any cause and infection after potential confounders.

**Results:** We linked clinical and administrative data for 1,767 persons with MS, most of whom were women living in urban areas. All individual indicators tested had specificities >90% for severe disability, and all but a diagnosis of visual disturbance had positive predictive values (PPV) >70%. The combination of home care or long-term care use or rehabilitation admission had a sensitivity of 61.9%, specificity of 90.76%, PPV of 70.06% and negative predictive of 87.21%. Based on regression modeling, the best-performing algorithm for predicting the EDSS as a continuous variable included age, home care use, long-term care admission, admission for rehabilitation, visual disturbance, other paralytic syndromes and spasticity. The mean difference between observed and predicted values of the EDSS was −0.0644 (95%CI −0.1632, 0.0304). Greater disability, whether measured using the clinical EDSS or either of the administrative data algorithms was similarly associated with increased hospitalization rates due to any cause and infection.

**Conclusion:** We developed and internally validated an algorithm for disability in MS using administrative data that may support population-based studies that wish to account for disability status but do not have access to clinical data sources with this information. We also found that more severe disability is associated with increased health care use, including due to infection.

## Introduction

Administrative claims data offer many advantages for epidemiologic and health services research. In Canada, and many other publicly funded health systems; they are population-based, accessible, and relatively low cost compared to primary data collection (1). They have been used to study the incidence and prevalence of multiple sclerosis (MS), as well as health services use and mortality. Despite their utility, administrative data suffer from limitations including that they are not collected for research purposes; so their validity for research use must be assessed (2). The data may also lack clinical details relevant to the proposed research questions.

Disability status is a highly relevant clinical characteristic which influences health care utilization and other important outcomes such as mortality in people with MS. Although case definitions have been validated for relapses (3, 4), and one study developed a predictive model for health care costs in MS (5), no clinically validated administrative case definition for disability exists in multiple sclerosis (MS) (5, 6). While linkage to clinical datasets can mitigate this deficiency, clinical datasets generally capture only a subset of the population encompassed by administrative data (7), inducing a potential selection bias. Disability is also a potential predictor or confounder of association. For example, disability is associated with the risk of infection-related hospitalizations (8) but some studies relying on administrative data have been unable to account for this factor (9). Thus a population-based, valid means of identifying disability in administrative data is needed.

We aimed to develop and internally validate an algorithm for disability status in MS using administrative (health claims) data, and to compare the association of disability status and health care use when employing a clinically assessed Expanded Disability Status Scale (EDSS) score vs. our preferred disability algorithms.

## Methods

### Setting

We conducted this study in the central Canadian province of Manitoba, which has a population of ~1.33 million persons. In Manitoba, health care is universal and publicly funded for medically necessary services. Health system contacts are captured in administrative databases. Specialty MS care, including prescriptions for disease-modifying therapies, is provided through one provincial MS Clinic and is captured in the MS Clinic Registry. Ethics approval was provided by the Health Research Ethics Board of the University of Manitoba. Approvals for data access were granted by the Health Information Privacy Committee and the Winnipeg Regional Health Authority.

### Data Sources

We used clinical and administrative data sources. The Winnipeg MS Clinic was established in 1998 and serves the entire province of Manitoba, delivering care to ~2,700 people with MS annually. All Manitobans who wish to receive provincially-funded disease-modifying therapies are re-evaluated at least annually. In May 2011, the MS Clinic established a registry; >2,100 persons are participating (89% of those approached) and have agreed to linkage of their clinical and administrative data for research purposes. The registry can be linked to the administrative dataset using the personal health identification number. The registry contains data on disability measured using the Expanded Disability Status Scale score (EDSS) (10) and the corresponding dates of assessment, recorded during routine MS clinic appointments by EDSS-certified neurologists. The EDSS is an ordinal measure of disability based on the neurological examination. Total EDSS scores range from 0 (no disability) to 10 (death due to MS), and are derived from scores on visual, brainstem, pyramidal, sensory, cerebellar, sphincter, and cerebral functional systems, as well as an observed walk of up to 500 meters. An EDSS score of 6.0 indicates the need for unilateral assistance to walk, while a score of 6.5 indicates the need for bilateral assistance, and 7.0 indicates the need for a wheelchair. EDSS scores of >5 are associated with impaired activities of daily living (11).

We used administrative datasets held in the Population Health Data Repository at the Manitoba Centre for Health Policy. When selecting the datasets to be used, we considered their utility for identifying individuals with disability and whether they were also available in other Canadian provinces to allow widespread application of our case definition. The datasets used included the population registry, Discharge Abstract Database (DAD), medical services (physician) claims, Drug Program Information Network (DPIN) database, National Rehabilitation Reporting system (NRS), long-term care database, home care database, and employment and income assistance (EIA)/Social Allowances Management Information (EIA/SAMIN). The information used from each dataset is described further below.

The population registry provided sex, dates of birth, death and health care coverage, and postal code. The DAD captures hospitalizations including dates of admission and separation. Before 2004, diagnoses were recorded using International Classification of Disease 9^th^ edition, clinical modification (ICD-9-CM) codes. From 2004 onward, they were recorded using ICD-10-Canadian version (CA). Medical services provided the date and type of service, and one physician-assigned diagnosis recording using a 3-digit ICD-9-CM code. DPIN records all community-dispensed prescriptions including the date, drug name and drug identification number (DIN). The NRS provided admissions for physical rehabilitation including admission dates and discharge dates. The long-term care database indicated the date of all personal care (nursing) home admissions. In Canada, such admissions are generally permanent rather than for rehabilitation purposes. Over 70% of persons with MS in Canadian long-term care settings use wheelchairs, two-thirds have bowel incontinence, and 43.5% have difficulty swallowing (12), making this setting a potentially specific indicator of disability. The home care database provided the dates for opening and closing of home care services which allowed the determination of the number of days with an open home care file. To be eligible for home care individuals must have limitations in their activities of daily living; 48% of persons with MS in this setting use a wheelchair and one-third have urinary incontinence (12). This database is population-based for individuals living in the Winnipeg Regional Health Authority, where 70% of the Manitoba population resides. EIA is available to Manitobans with no other means of financial support. Individuals with a mental or physical disability likely to last >90 days and which prevents the individual from earning enough money to money for basic financial needs are eligible to apply. An indicator of physical disability can be recorded with values of hearing, illness, mobility/coordination-no wheelchair, mobility/coordination-wheelchair, other, speech and vision. These datasets were linked using an encrypted unique identifier to crate the administrative dataset. Using postal code, this dataset was linked to census data from Statistics Canada to determine household income of each individual's enumeration area (13), as a measure of socioeconomic status (SES).

### Study Population

Using hospital, physician and prescription claims for the period April 1, 1984 to March 31, 2017, we identified Manitobans with MS using a validated case definition (14, 15). This case definition required an individual to have at least three health care encounters for MS, and has a sensitivity of 99.5%, specificity of 98.5% and positive predictive value of 99.5%. The date of the first demyelinating disease claim (e.g., optic neuritis) was designated as the index date (Supplementary Table 1) (14). This constituted the prevalent MS cohort. For the development of the disability case definition we limited this prevalent cohort to adults aged ≥18 years, alive and living in Manitoba during the period April 1, 2014 to March 31, 2017. This reflected the most recent data available at the time the study was approved. We then linked this cohort to data from the MS Clinic Registry for consenting participants. This constituted the “disability” cohort.

### Health Care Utilization Outcomes

Health care use outcomes included: annual ambulatory physician visit rates and annual hospitalization rates due to any cause; and due to infection specifically. Infections were identified using ICD-9/ICD-10 codes listed in Supplementary Table 2, consistent with prior research in British Columbia, Canada (9).

### Covariates for Health Care Utilization Analysis

Covariates included sex (female as reference group), current age (continuous), age at the index date (continuous), year of diagnosis (1984–1989 [reference], 1990–1994, 1995–1999, 2000–2004, 2005–2009, 2010 onward), SES, region, comorbidity, and use of disease-modifying therapy. We categorized SES into quintiles (lowest quintile of SES as reference group) at the index date. Regions were classified as urban (Winnipeg, population >600,000 and Brandon, population >47,000) or rural. Comorbidity was captured using a modified version of the Charlson score (categorized as 0, 1, ≥2) to avoid misclassifying symptoms or signs of MS as comorbidities (16). MS disease-modifying therapy (DMT) use was identified using drug identification numbers (DINs) for therapies available during the study period (Supplementary Table 3 and was classified as any vs. none, updated annually.

### Disability Indicators and Algorithms

#### Indicators

We created a series of candidate indicators using the administrative data. These included indicators based on use of particular health care services, use of specific diagnostic codes, and codes based on EIA. Indicators based on the use of particular health care services included: (i) an open home care file for >90 days; (ii) long-term care (residence in a nursing home); and (iii) discharge following a rehabilitation admission. Indicators based on the presence of ≥2 ICD-9/ICD-10-CA codes ≥90 days apart included those related to: visual disturbance; abnormalities of gait and mobility; speech disturbances not elsewhere classified; limitation of activities due to disability; need for assistance due to reduced mobility; hemiplegia and hemiparesis; quadriplegia and quadriparesis; wheelchair dependence; falls; dysphagia; dysarthria; spasticity; incontinence; pressure ulcer; dementia in other diseases; malaise and fatigue; skin sensation disturbance (Supplementary Table 1). These codes were selected to reflect symptoms or signs of MS that might be captured in the EDSS, or secondary conditions that occur more often in individuals with severe disability due to MS. The requirement for two codes over an interval was designed to ensure that the symptoms were persistent, and not transient due to a relapse. In a sensitivity analysis we altered the intervals between diagnosis codes to ≥60 days or ≥120 days. Indicators based on use of social services, which would only be available only in Manitoba, included use of EIA because of mobility/coordination issues or another physical disability code.

#### Algorithms

The goal was to develop two algorithms based on combinations of these indicators. The first algorithm would be to predict severe disability, given that this has been reported as easier to accomplish than predicting all levels of disability in the general population (17). The second algorithm would describe disability as a continuous measure, and could be categorized as needed. The approach to algorithm development is articulated further below.

## Analysis

We summarized the characteristics of study cohorts using descriptive statistics including mean [standard deviation (SD)], median [interquartile range (IQR)], and frequency (percent) as appropriate. We report standardized differences for comparisons between groups; 0.20 represents a small effect size.

### Algorithms for Severe Disability as a Dichotomous Variable (Disability-Sev)

We randomly split the “disability” cohort into discovery and validation cohorts. For each of the indicators we identified the closest EDSS score in time. Due to small numbers of individuals with some of the indicators we allowed the interval between the measurement of the EDSS score and subsequent assessment of the indicator to be up to 365 days. In a complementary analysis we allowed EDSS measurements to occur 365 days before or after the measurement of the indicator. For individuals who did not have a particular indicator and therefore no time point of reference we used the EDSS score closest to the study midpoint. Then we reported the minimum, maximum and median (IQR) EDSS scores at that time. Based on these findings, and the proportion of scores which could be classified as mild (0–3.0), moderate (3.5–5.5) and severe (6.0–9.5) (18), we subsequently categorized disability as severe (EDSS 6.0–9.5) or not severe (EDSS <6.0) and used this as the reference standard for assessing performance of the case definitions. For each indicator, we determined the sensitivity, specificity, positive predictive value (PPV) and negative predictive values (NPV), and agreement (as measured using a kappa statistic) (19) as compared to the severe disability variable. We conducted this analysis in the discovery cohort, then repeated it in the validation cohort, as a means of internal validation; we report the findings in the validation cohort. Our goal was a PPV ≥ 70% (20).

Since algorithms designed in this fashion may not capture interactions between component indicators and non-linear effects, we also applied statistical approaches to see if superior case definitions could be identified. After considering several approaches including classification trees, random forests and support vector machines we opted to use logistic regression because it has the greatest clinical interpretability. Logistic regression models the linear association between the variables of interest and the log-odds of the outcome, in this case severe disability. Initially, we considered all possible indicators. We also included age (continuous variable) since it is strongly associated with disability severity in MS (21). As models are more data intensive for this analysis we used the entire sample and used bootstrapping for internal validation and to avoid overfitting related to testing multiple models in a small sample (22). Specifically, we trained the logistic regression on the entire sample and then recorded the area under the receiver operating characteristic curve (AUC, referred to as the naïve C-statistic). We then drew 500 bootstrap samples with replacement from the original sample and trained the same logistic model on each sample to obtain 500 AUCs (C-statistics). The mean of the differences between the original AUC (naïve C-statistic) and those computed from each of the bootstrap samples is known as optimism. The difference between optimism and the naïve C-statistic is called the optimism-corrected C-statistic (23). This statistic gives an idea of how the model would perform in a dataset that it was not trained on. Given the unbalanced distribution with respect to severe disability, we also repeated these models in which we randomly under-sampled the larger class. This can improve performance of classification algorithms (24), and may produce superior performance to other methods. We assessed model discrimination using the AUC, classification accuracy, sensitivity, specificity, positive and negative predictive value.

### Algorithms for Disability as a Continuous Measure (Disability-Cont)

Given the bounded distribution of the EDSS (0–10), and to ensure that predicted values did not fall outside the range of possible values we used truncated regression models to predict the EDSS. Because an EDSS of 10 indicates death, and we were interested in disability status among living individuals we set the upper bound as 9.5. The variables used in this approach were based on those selected using the bootstrap-validated logistic regression of the Disability-Sev outcome, given the high values of the naïve C-statistic and the optimism-corrected C-statistic. We assessed assumptions of normality using quantile-quantile plots. We assessed overall model fit using a pseudo-R^2^ estimated as the squared correlation of the observed and predicted values of the EDSS (25), and mean square error. We also report the mean difference between the observed and expected values, and used Bland-Altman plots to further evaluate agreement between the observed and expected values (26).

Relapses may be associated with transient increases in EDSS. Our clinical dataset lacked information regarding relapses but we applied a previously validated case definition for severe (treated) relapses, and repeated our regression analyses for algorithm development after excluding affected individuals as a sensitivity analysis (4).

### Applying the Disability Algorithms

After we selected our preferred algorithms for disability, we tested their association with health care use, using the entire linked clinical-administrative dataset. First, we report annualized hospitalization rates and physician visits and overall, and due to infection.

Second, we tested the association between severe disability, based on the clinically recorded EDSS (hereinafter c-EDSS, updated annually), and hospitalization rates using zero-inflated Poisson regression models using generalized estimating equations (GEE) to account for dependence of observations within individuals. We modeled physician visits using negative binomial regression model with GEE. Both models included as an offset the number of person-days the patient was resident in MB, to account for differing follow-up time between individuals. Covariates included sex, age, SES (updated annually at the beginning of the fiscal year), year of diagnosis, disease duration, comorbidity (updated annually at the beginning of the fiscal year), and use of disease-modifying therapy as previously defined. Then, we repeated the analysis substituting the preferred algorithm for severe disability (*Disability-Sev*) for the c-EDSS dichotomized at 6.0, expecting to find similar associations between the c-EDSS and health care use as between the disability algorithms and health care use. We used a similar approach to testing the second algorithm for disability (*Disability-Cont*) in which we included the c-EDSS as continuous then substituted Disability-Cont for c-EDSS. Finally, we categorized the Disability-Cont as mild (0–3.0), moderate (>3.0 and ≤5.5) and severe (>5.5–9.5). We report rate ratios (RR) and 95%CI for the observed associations.

Statistical analyses used SAS V9.4 (SAS Institute Inc., Cary, NC).

## Results

### Study Sample

We identified 3,703 individuals with MS who were alive and living in Manitoba during the study period. Of these, 1767 had linkable clinical data. The characteristics of the linked and unlinked cohorts are shown in Table 1. Most individuals with MS were women and two-thirds lived in urban areas. The median number of EDSS scores available during the study period was three. Among the 1,419 participants with more than one EDSS score during the study period, 747 (52.6%) had the same score at all time points, and 298 (21.0%) had a subsequent EDSS score within ±0.5 of the prior EDSS score. At the beginning of the study period 392 (22.2%) of participants had an EDSS score ≥6.0. Overall, 143 (8.1%) individuals were treated for 204 relapses during the study period.

**Table 1:** Characteristics of study participants.

**Characteristic**	**“Disability” Cohort (*n* = 3,703)**	**Linked** **(*n* = 1767)**	**Unlinked (*n* = 1936)**	**Std Diff**
Female, *n* (%)	2,709 (73.2)	1,297 (73.4)	1,412 (72.9)	0.01
Age at MS diagnosis, mean (SD)	38.5 (11.0)	37.3 (10.7)	39.6 (11.3)	0.15
Duration of follow-up from the index date (years), median (IQR)
Urban region of residence, *n* (%)	2,417 (65.3)	1,143 (64.7)	1,274 (65.8)	0.02
Socioeconomic status, *n* (%)
Quintile 1 (lowest)	558 (15.1)	252 (14.3)	306 (15.8)	0.04
Quintile 2	692 (18.7)	350 (19.8)	342 (17.7)	0.05
Quintile 3	823 (22.2)	397 (22.5)	426 (22.0)	0.01
Quintile 4	761 (20.6)	347 (19.6)	414 (21.4)	0.04
Quintile 5 (highest)	842 (22.7)	416 (23.5)	426 (22.0)	0.04
Number of EDSS scores, median (IQR)	-	3 (2, 3)	-	
EDSS	-		-	
Mean (SD)		3.74 (2.55)		
Median (Min-Max)		3 (0–9.5)		
≥6.0		392 (22.2%)		
Open home care file >90 days, *n* (%)
Ever	884 (23.9)	407 (23.0)	447 (24.6)	0.04
Study period	528 (14.3)	254 (14.4)	274 (14.2)	0.01
Admission to personal care home, *n* (%)
Ever	214 (5.8)	66 (3.7)	148 (7.6)	0.17
Study period	64 (1.7)	32 (1.8)	32 (1.7)	0.01
Use of Employment Income Assistance, *n* (%)
Ever	559 (15.1)	215 (12.2)	344 (17.8)	0.16
Study period^a^	268 (7.2)	91 (5.2)	177 (9.1)	0.15
Admitted for rehabilitation at any time in study period, *n* (%)
Ever	216 (5.8)	105 (5.9)	111 (5.7)	0.01
Study period	42 (1.1)	21 (1.2)	21 (1.1)	0.01
Diagnosis codes ≥2 codes ≥90* days apart during study period, *n* (%)
Visual disturbance	343 (9.3)	151 (8.6)	192 (9.9)	0.04
Blindness and low vision	37 (1.0)	15 (0.85)	22 (1.1)	0.03
Abnormalities of gait and mobility	s	0 (0)	s	
Speech disturbances not elsewhere classified	0 (0)	0 (0)	0 (0)	0
Limitation of activities due to disability	0 (0)	0 (0)	0 (0)	0
Need for assistance due to reduced mobility	0 (0)	0 (0)	0 (0)	0
Hemiplegia and hemiparesis	36 (0.97)	12 (0.68)	24 (1.24)	0.06
Other paralytic syndromes/Quadriplegia and quadriparesis	300 (8.1)	166 (9.4)	134 (6.9)	0.09
Falls	61 (1.6)	10 (0.57)	51 (2.6)	0.16
Dementia in other diseases classified elsewhere	9 (0.24)	0 (0)	9 (0.46)	0.10
Incontinence–urinary or fecal	27 (0.73)	12 (0.68)	15 (0.77)	0.01
Malaise and fatigue	73 (2.0)	35 (2.0)	38 (1.96)	0.00
Pressure ulcer	38 (1.0)	17 (0.96)	21 (1.08)	0.01
Spasticity	37 (1.0)	26 (1.5)	11 (0.57)	0.09
Skin sensation disturbance	14 (0.38)	s	s	0.03
Dysarthria	0 (0)	0 (0)	0 (0)	0
Dysphagia	0 (0)	0 (0)	0 (0)	0

a *203/268 and 64/68 had a disability code associated with their use of Employment Income Insurance (hearing, illness, mobility/coordination-no wheelchair, mobility/coordination-wheelchair, other, speech and vision); s = suppressed cell sizes <5 to protect privacy and confidentiality*.

### Use of Health Services and Diagnostic Indicators

During the study period nearly 15% used home care services, fewer than 10% used EIA, and fewer than 2% were admitted for rehabilitation or were admitted to long-term care (Table 1). The proportion of individuals using these services was higher when we considered whether they were ever used, that is, if they were used before or during the study period. The median (IQR) EDSS for individuals using home care services was 6.5 (6, 7.5), admitted to long-term care was 7 (6.5, 8), and discharged after a rehabilitation admission was 6.5 (4, 7.5). Although an indicator of physical disability can be recorded in the EIA dataset this information was missing for nearly half of individuals (49.3%), and the mobility codes which would map well to the c-EDSS were only reported for 5.6% of individuals ever using EIA. Therefore, we did not move forward with using this indicator. Most of the diagnostic codes for symptoms commonly associated with MS and captured in some way in the c-EDSS were used infrequently or not all. Only diagnoses of visual disturbance and other paralytic syndromes were used in over 5% of the members of the linked and unlinked cohorts during the study period. These findings did not change when we altered the intervals between diagnosis codes to ≥60 days or ≥90 days (Supplementary Table 4). The median (IQR) for individuals with visual disturbance codes was 2 (1.5, 4), and for other paralytic syndromes was 6.25 (5.5, 7.5).

### Algorithms for Severe Disability (Disability-Sev)

All of the individual indicators tested had specificities exceeding 90% for severe disability (c-EDSS ≥6.0), and all but one (visual disturbance) had PPV exceeding the 70% threshold (Table 2). Negative predictive values exceeded 70% but sensitivities were generally low; the highest sensitivity was for home care use (58.2%). Combining indicators improved sensitivity at the expense of lower specificity and lower PPV. For example, the combination of home care or long-term care use or rehabilitation admission had a sensitivity of 61.9%, specificity of 90.76%, PPV of 70.06% and NPV of 87.21%. Findings were similar when we used indicators requiring diagnostic codes to be ≥60 days apart (Supplementary Table 5). Findings were similar when we allowed c-EDSS to be measured within 365 days before or after the indicator (data not shown).

**Table 2:** Performance of disability indicators for severe disability measured within 365 days after EDSS record, and within study window.

**Indicator**	**Sens (95%CI)**	**Spec (95%CI)**	**PPV (95%CI)**	**NPV (95%CI)**	**Kappa (95%CI)**
Open home care file >90 days	58.2 (50.82, 65.32)	92.42 (89.86, 94.51)	72.85 (65.02, 79.76)	86.36 (83.29, 89.05)	0.54 (0.47, 0.61)
Long-term care (Nursing home) admission	7.58 (4.30, 12.19)	99.82 (98.99, 100.0)	93.75 (69.77, 99.84)	74.97 (71.66, 78.07)	0.10 (0.05, 0.16)
Discharged after rehabilitation admission	15.38 (10.63, 21.23)	97.81 (96.21, 98.86)	71.43 (55.42, 84.28)	76.46 (73.14, 79.56)	0.18 (0.11, 0.24)
Visual disturbance	6.97 (3.86, 11.41)	91.94 (89.33, 94.08)	24.14 (13.87, 37.17)	72.86 (69.37, 76.15)	0 (0, 0.04)
Other paralytic syndromes	25.50 (19.61, 32.13)	96.34 (94.40, 97.75)	71.83 (59.90, 81.87)	77.93 (74.60, 81.00)	0.27 (0.20, 0.35)
Falls	2.46 (0.80, 5.65)	99.82 (98.99, 100.0)	83.33 (35.88, 99.58)	73.46 (70.13, 76.60)	0.03 (0.00, 0.06)
Home care OR nursing home	59.38 (52.06, 66.39)	92.25 (89.67, 94.36)	73.08 (65.40, 79.86)	86.51 (83.45, 89.18)	0.55 (0.48, 0.62)
Home care OR nursing home OR discharged after rehabilitation admission	61.90 (54.57, 68.86)	90.76 (88.00, 93.06)	70.06 (62.5, 76.89)	87.21 (84.17, 89.86)	0.55 (0.48, 0.62)
Home care OR nursing home OR other paralytic syndromes or falls	64.58 (57.37, 71.34)	88.91 (85.96, 91.43)	67.39 (60.11, 74.11)	87.61 (84.56, 90.25)	0.54 (0.47, 0.61)
Home care OR nursing home OR discharged after rehabilitation admission OR other paralytic syndromes	66.67 (59.46, 73.34)	87.62 (84.54, 90.27)	65.29 (58.11, 71.98)	88.27 (85.24, 90.87)	0.54 (0.47, 0.61)
Home care OR nursing home OR discharged after rehabilitation admission OR other paralytic syndromes or falls	66.67 (59.46, 73.34)	87.62 (84.54, 90.27)	65.29 (58.11, 71.98)	88.27 (85.24, 90.87)	0.54 (0.47, 0.61)
Home care OR nursing home OR discharged after rehabilitation admission OR other paralytic syndromes or falls or hemiplegia or wheelchair dependence or pressure ulcer	67.20 (60.01, 73.84)	87.62 (84.54, 90.27)	65.46 (58.32, 72.13)	88.43 (85.42, 91.02)	0.54 (0.47, 0.61)

*Sens, sensitivity; spec, specificity; PPV, positive predictive value; NPV, negative predictive value*.

When we applied the logistic regression approach several indicators were relevant for prediction, including home care use, long-term care admission, rehabilitation admission, visual disturbance and spasticity. As the findings from logistic model most readily translate into an interpretable algorithm that can be applied in future studies we present those findings. Based on the preferred logistic model (see Table 3, Model A), at a predicted probability ≥0.25 the sensitivity for c-EDSS ≥6.0 was 49%, specificity 93.8%, PPV 72%, and NPV 85%. The naïve C-statistic for this model was 0.844 with an optimism value of 0.0024 and optimism-corrected C-statistic of 0.842. After excluding individuals with a treated relapse, the regression equations were similar (Supplementary Table 6).

**Table 3:** Logistic regression-based algorithm for severe disability (regression coefficients shown)^*^.

**Model**	**Intercept**	**Age**	**Home Care**	**Long-term care**	**Rehabilitation**	**Visual disturbance**	**Other paralytic syndromes**	**Spasticity**	**C-statistic**	**Optimism-corrected C-statistic**	**AIC**
A	−3.8998	0.0488	1.9745	1.17396	1.4058	−1.2239	0.9635	2.22194	0.844	0.842	1281.4
B	−3.9934	0.0494	1.9535	1.6629	1.3796		0.9617	2.0995	0.840	0.839	1296.4
C	−3.9023	0.05	2.0974	1.7652		−1.1823	1.0907	2.2644	0.838	0.836	1306.0

* *Requires calculating probability of severe disability, where probability ≥0.25 indicates severe disability, AIC, Akaike's Information Criterion*.

### Algorithms for Disability (Continuous)

Based on our truncated regression models, the best-performing model for predicting the EDSS as a continuous variable included age, home care use (ever), long-term care admission (ever), hospitalization for rehabilitation (ever), visual disturbance, other paralytic syndromes and spasticity (Table 4). This model explained 40% of the variation in EDSS scores, and the mean difference between observed and predicted values of the EDSS was −0.0644 (95%CI −0.1632, 0.0304). The model tended to overestimate EDSS scores at the lower end of the range as evidenced by the model intercept which exceeded 0. The Bland-Altman plot is shown in Supplementary Figure 1, and shows some outliers. After excluding individuals with a treated relapse, the regression equations were similar (Supplementary Table 7).

**Table 4:** Truncated regression-based algorithms for disability as a continuous variable.

**Model**	**Intercept**	**Age**	**Home Care**	**Long-term care**	**Rehabilitation**	**Visual disturbance**	**Other paralytic syndromes**	**Spasticity**	**Mean Diff**	**MSE**	**Pseudo-R^**2**^**
A^a^	1.272558	0.040501	2.602358	1.427529	1.066686	−0.240803	1.355134	2.035198	−0.0644 (−0.1632, 0.0304)	2.09	40.4%
B	1.215997	0.041938	2.743066	1.420044			1.477834	2.152465	−0.0673 (−0.1649, 0.0303)	2.11	39.5%
C	1.24794	0.041645	2.741529	1.43335		−0.233042	1.475601	2.168455	−0.0672 (−0.1647, 0.0304)	2.10	39.6%

a *Did not improve adding non-linear term for age, all p < 0.0001. MSE, Mean standard error*.

### Association of Disability With Health Care Use

Using the linked clinical dataset, annual rates of physician visits and hospitalizations for any cause, and specifically due to infection over the study period are shown in Supplementary Table 8. Overall, 376 (21.3%; 95%CI: 19.4–23.2%) of individuals was hospitalized at least once, and 117 (6.6%; 95%CI: 5.5–7.8%) were hospitalized for infection at least once. The mean (SD) number of physician visits over the study period was 24.2 (18.9) for any reason, and 1.4 (2.3) for infection; 878 (49.7%) of individuals had at least one physician visit for infection.

**Figure 1:**
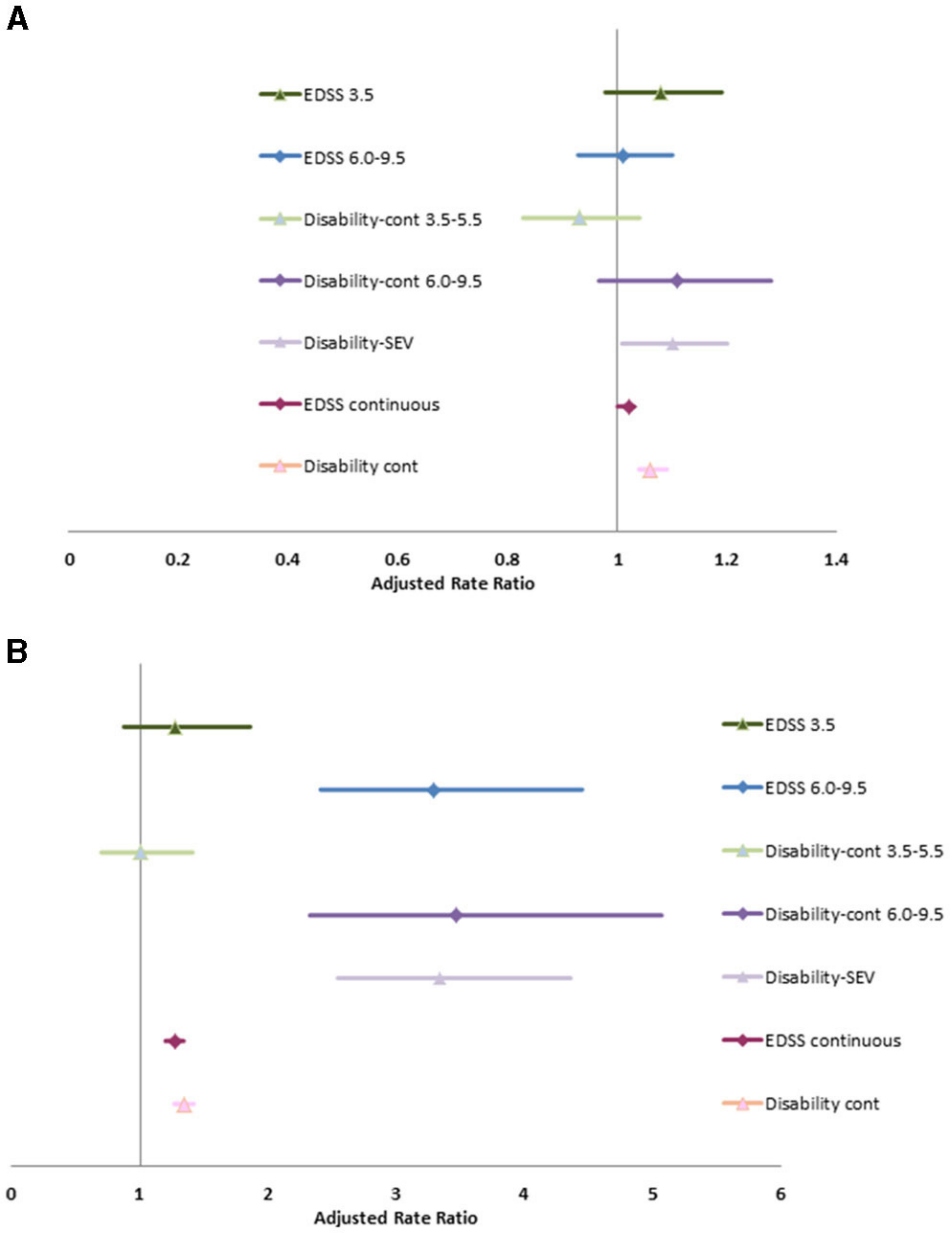
Association of disability status with physician visits **(A)** and hospitalizations **(B)**.

Without adjusting for covariates, severe disability (clinical EDSS ≥6.0) was associated with increase rates of physician visits (RR 1.12; 95%CI: 1.02, 1.22). After adjusting for age at diagnosis, diagnosis year, sex, SES, Charlson comorbidity score, disease duration and use of DMT, the association between severe disability and physician visits was attenuated for any reason or for infection, but was associated with an increased rate of hospitalizations due to any cause and due to infection (Supplementary Table 9, Figure 1). The associations of hospitalizations due to infection with severe disability are extreme with broad confidence intervals due to the small number of events. After adjusting for covariates, as a continuous variable, the c-EDSS was also associated with an increased rate of hospitalizations and physician visits due to any cause and due to infection.

When we substituted the Disability-SEV or Disability-Cont into the model, the associations between disability and health care use were similar. Similarly, when we substituted the categorized version of Disability-Cont into the model the findings remained similar to those when mild, moderate and severe c-EDSS categories were included.

## Discussion

Administrative health claims data in many jurisdictions lack key clinical information, such as disability status. We developed and validated case definitions for disability in individuals with MS using a combination of age, diagnostic codes, and use of specific health care services including home care, long-term care and hospitalization for rehabilitation. Unlike other efforts to develop claims-based indicators of disease severity for MS we did not limit our study population to individuals under age 65 years, nor to those using disease-modifying therapy. We used a truncated linear regression model to generate a predicted EDSS score, with a proportion of variation explained of 40%. In keeping with prior work in the general population (17), we also developed an indicator for severity disability. As compared to mild disability based on the EDSS, severe disability was associated with elevated rates of hospitalization and physician visits due to any cause. Findings were similar when we substituted disability based on our algorithms.

Other efforts have been made to develop claims-based indicators (definitions) of disease severity in MS but have all used datasets from the United States, limited their study populations to individuals aged 18–64 years, and none validated them against a clinical reference standard. The first used commercial claims data from the United States for individuals with MS who were treated with disease-modifying therapies (5). Components of the indicator included diagnosis codes for rehabilitation services, altered mental status, pain, disability, stiffness, balance, urinary incontinence, numbness, malaise/fatigue, and infections, and it was developed to predict health care costs. Cost predictions were most accurate for individuals in the lowest tercile of severity. Due to the emphasis on costs, this indicator may not perform well in other health care systems. Another effort also used commercial claims data in the United States and developed an indicator relying on diagnostic codes, prescription claims and procedure codes to categorize individuals with MS aged 18–64 years according to disease severity (6). The investigators focused on several symptoms, specifically bowel/bladder function (mainly incontinence), cognitive function, psychiatric disorders, and physical function (spasticity, paresthesias coupled with use of pain medications, cane/walker, or a prescription for fampridine). Individuals were classified as having mild, moderate or severe MS. They showed that individuals with more severe MS had a greater comorbidity burden and higher medical costs. A third effort also relied on diagnostic codes for several symptoms, use of durable medical equipment, and use of disease-modifying therapy; it similarly classified individuals as having mild, moderate or severe disease and used health care costs as a reference standard. The severity measure and health care costs were strongly associated with each other (27). The utility of this claims-based definition of disease severity was subsequently assessed in a Medicare-eligible population; the disability-eligible Medicare population had a higher severity score than the age-eligible Medicare population and an increased risk of hospitalization (28).

Efforts to develop indicators of the severity of disability in the general population have also been limited. In 2020, a systematic review identified two algorithms aimed at identifying reproductive-aged women with physical and sensory disabilities in administrative data. Both algorithms were developed in the United States (29). The first algorithm, the Access Risk Classification System (ARCS), identified disability based on the need for assistance or accommodation when accessing health care as identified based on ICD-9 diagnostic codes, procedure codes and prescription claims (17), some of which were not available in our data. The ARCS was validated based on self-reported disability status and classified a low proportion of individuals correctly. It performed best at identifying individuals with likely complex medication needs, and this observation informed our decision to explicitly develop a case definition for severe disability. The second algorithm relied on the presence of diagnostic codes for conditions associated with mobility limitations, including MS, and Current Procedural Terminology (CPT) codes for assistive devices including canes, walkers and wheelchairs available in Medicare data (30). Validation of this algorithm was limited to an assessment of the face validity of the codes used.

Comparisons between cohorts with and without MS have shown that cohorts with MS are at increased risk of hospitalization due to any cause (31), and due to infection (9, 32). Within the MS population, a study in Finland found that individuals hospitalized for infection had a higher median level of disability than individuals who were not (8). Similarly, an American study that compared infection-related hospitalizations in Veterans with and without MS found that using a wheelchair or walker was associated with an increased risk of hospitalization (33). Our findings, whether we used the clinically measured EDSS or our disability algorithm, are concordant with these observations. The consistency of the findings when we substituted the disability algorithm for the clinically scored EDSS also provides further support for the utility of our algorithm.

Strengths of this study included the emphasis on population-based data sources available in Canadian other jurisdictions to enhance the applicability of our case definition. The clinical dataset and administrative datasets were collected prospectively and independently of each other limiting ascertainment bias. Also, we used traditional and regression-based methods of developing algorithms. We internally validated our findings using training and validation cohorts, or bootstrapping approaches. Our algorithms use data available irrespective of whether the individual has MS or not, thus supporting comparative studies that may wish to account for disability status across populations as the criteria for access to home care, long-term care and rehabilitation services do not depend on diagnosis. Limitations of this study should also be considered. We allowed an interval of up to 365 days between EDSS scores and the administrative data indicators to maximize sample size. This could have reduced model accuracy due to changes in the clinical status of the participant, but 73% of participants had EDSS scores which did not change or varied within ±0.5 of each other. Similarly, we could not determine what EDSS scores were associated with relapses, but our sensitivity analyses excluding individuals with treated relapses produced similar findings to the primary analysis. The home care dataset we used covers the Winnipeg Regional Health Authority where 70% of Manitobans reside. The lack of coverage outside this health region would have reduced the sensitivity of home care as an indicator of severe disability. In addition, individuals with MS who have severe disability but family who could meet their needs without home care or long-term care support may also be missed unless they are captured by another indicator. Equipment codes for devices such as canes and walkers are not available in Manitoba but in other jurisdictions may be helpful to capture such individuals. We did not incorporate prescription claims because these are not available in several Canadian provinces and in some non-Canadian jurisdictions, but medications for neurogenic bladder, spasticity or mobility might have improved sensitivity since our findings suggest that diagnostic codes for MS-related symptoms are not used frequently. While our linked dataset included over 1,700 individuals this is a relatively modest sample size for development and testing of a complex case definition. We did not have an independent dataset to support external validation of the case definition, therefore the performance of our indicators and algorithms should be tested in other jurisdictions to establish generalizability.

## Conclusion

In summary, we developed and validated an algorithm for disability in MS using administrative data that may support population-based studies that wish to account for disability status but do not have access to clinical data sources with this information. We also found that more severe disability is associated with increased health care use, including hospitalizations due to infection, supporting the importance of vaccinations and other preventive health measures for infection.

## Data Availability Statement

The data analyzed in this study is subject to the following licenses/restrictions: data used in this article were derived from administrative health data as a secondary use. The data were provided under specific data sharing agreements only approved for use at the Manitoba Centre for Health Policy. The original source data are not owned by the researchers or Manitoba Centre for Health Policy and as such cannot be provided to a public repository. Where necessary, source data specific to this article or project may be reviewed at MCHP with the consent of the original data providers along with the required privacy and ethical review bodies. Requests to access these datasets should be directed to Charles Burchill, (Charles_Burchill@cpe.umanitoba.ca).

## Ethics Statement

The studies involving human participants were reviewed and approved by University of Manitoba Health Research Ethics Board. Written informed consent for participation was not required for this study in accordance with the national legislation and the institutional requirements.

## Author Contributions

RM and JM conceived of the idea. QT and OE conducted the statistical analyses. RM drafted the manuscript. All authors revised the manuscript and approved of the final version.

## Funding

This study was funded in part by a Manitoba Research Chair (to RM). RM is supported by the Waugh Family Chair in Multiple Sclerosis. The funding source(s) had no role in the study design, collection, analysis or interpretation of the data, nor in the decision to submit the article for publication.

## Conflict of Interest

RM receives research funding from: CIHR, Research Manitoba, Multiple Sclerosis Society of Canada, Multiple Sclerosis Scientific Foundation, Crohn's and Colitis Canada, National Multiple Sclerosis Society, CMSC and the US Department of Defense, and is a co-investigator on studies receiving funding from Biogen Idec and Roche Canada. JM receives research funding from the Multiple Sclerosis Scientific Foundation, Research Manitoba and Roche Canada. The remaining authors declare that the research was conducted in the absence of any commercial or financial relationships that could be construed as a potential conflict of interest.

## Publisher's Note

All claims expressed in this article are solely those of the authors and do not necessarily represent those of their affiliated organizations, or those of the publisher, the editors and the reviewers. Any product that may be evaluated in this article, or claim that may be made by its manufacturer, is not guaranteed or endorsed by the publisher.
