# Supplementary material for: Development and Internal Validation of a Disability Algorithm for Multiple Sclerosis in Administrative Data
Source: Front Neurol. 2021 Nov 2;12:754144. doi: 10.3389/fneur.2021.754144 (PMC8592934; doi:10.3389/fneur.2021.754144)
Supplement: Supplementary file 1 [file Data_Sheet_1.docx]

Table e1. Diagnostic codes for demyelinating disease

| - **Condition** | - **ICD-9-CM^a^** | - **ICD-10-CA code^a^** |
| --- | --- | --- |
| - Optic neuritis | - 377.3 | - H46 |
| - Transverse myelitis | - 323.82 | - G37 |
| - Acute disseminated encephalomyelitis | - 323 | - G36.9 |
| - Demyelinating disease of CNS unspecified | - 341.9 | - G37.8 |
| - Other acute disseminated demyelination | - G36 | - G36 |
| - Multiple sclerosis | - 340 | - G35 |
| - Visual disturbance | - 368, 269 | - H53.2, H53.4, H54 |
| - Abnormalities of gait and mobility | - 781.2, 719.70 | - R26 |
| - Speech disturbances not elsewhere classified | - 784.59 | - R47.1 |
| - Limitation of activities due to disability |  | - Z73.6 |
| - Need for assistance due to reduced mobility |  | - Z74.0 |
| - Hemiplegia and hemiparesis | - 342 | - G81 |
| - Quadriplegia and quadriparesis | - 344 | - G82 |
| - Wheelchair dependence | - V46.3 | - Z99.3 |
| - Falls | - E888, E9177, E9178, E9293 | - W01, W05, W06-W19 |
| - Dysphagia | - 787.22, 787.23 | - R13.12, R13.13 |
| - Dysarthria | - 784.51 | - R47.1 |
| - Spasticity | - 781.0 | - R252. |
| - Incontinence | - 788.3, 787.6 | - R15, R32 |
| - Pressure ulcer | - 707.00 | - 707.09 |
| - Dementia in other diseases | - 294.1 | - F02.0 F03 |
| - Malaise and fatigue | - 780.7, 799.3 | - R53, G933 |
| - Skin sensation disturbance | - 782.0 | - R20 |

1. International Classification of Diseases (ICD), 9^th^ revision, Clinical Modification (ICD-9-CM) (340) and ICD 10^th^ revision, Canadian version (ICD-10-CA)

Table e2. ICD-9-CM and ICD-10-CA codes for identification of infection-related hospital admissions and physician visits

| Intestinal infectious diseases |  | 001.xx – 009.xx | A00.xx -A09.xx |
| --- | --- | --- | --- |
| Tuberculosis |  | 010.xx – 018.xx | A15.xx -A19.xx |
| Zoonotic bacterial diseases |  | 020.xx – 027.xx | A20.xx -A28.xx |
| Other Bacterial Disease (eg. Leprosy Diphtheria Septicemia, Diseases due to other mycobacteria, Septicemia) |  | 030.xx – 041.xx | A30.xx -A49.xx |
| HIV |  | 042.xx | B20.xx |
| Poliomyelitis and other Non-arthropod Borne Viral Disease of CNS |  | 045.xx – 049.xx | A80.xx -A81.xx, A85.xx -A89.xx |
| Viral Diseases Accompanied By Exanthem (eg. Small Pox; Cowpox and paravaccinia, Herpes Zoster, Herpes Simplex, Measles, Rubella) |  | 050.xx -059.xx | B00.xx -B09.xx |
| Arthropod-borne Viral Diseases |  | 060.xx – 066.xx | A83.xx -A85.xx, A90.xx -A99.xx |
| Other disease due to Viruses and Chlamydiae |  | 070.xx – 079.xx | B15.xx -B19.xx, A70.xx -A74.xx, A82.xx, B25.xx -B34.xx, B07.xx |
| Rickettsioses and other Arthropod-borne Disease |  | 080.xx – 088.xx | A75.xx -A79.xx, B50.xx -B57.xx |
| Syphilis and other Veneral Diseases |  | 090.xx – 099.xx | A50.xx -A64.xx |
| Other Spirochetal Diseases |  | 100.xx – 104.xx | A65.xx -A69.xx |
| Mycoses |  | 110.xx – 118.xx | B35.xx -B49.xx |
| Helminthiasis |  | 120.xx – 129.xx | B65.xx -B83.xx |
| Other Infections and Parasitic Diseases |  | 130.xx – 136.xx | B58.xx -B64.xx, B85.xx -B89.xx |
| Late Effects of Infectious and Parasitic Diseases |  | 137.xx – 139.xx | B90.xx -B94.xx |
| Bacterial Meningitis |  | 320.xx | G00.xx, G01.xx, G04.2x |
| Meningitis due to other organisms |  | 321.xx | B45.1x, G02.xx, |
| Meningitis of unspecified cause |  | 322.xx | G03.0x G03.8x, G03.1x, G03.9x |
| Intracranial and intraspinal abscess |  | 324.xx | G06.xx |
| Late effects of intracranial abscess or pyogenic infections |  | 326.xx | G09.xx |

Table e2: ICD-9-CM and ICD-10-CA codes for identification of infection-related hospital admissions and physician visits (continued)

| **Infection** |  | **ICD9** | **ICD10** |
| --- | --- | --- | --- |
| Blepharitis |  | 373.0x | H01.0x |
| Infective dermatitis of eyelid of types resulting in deformity |  | 373.4x | H01.8x |
| Other infective dermatitis of eyelid |  | 373.5x | H01.8x |
| Parasitic infection of eyelid |  | 373.6x | B89.xx |
| Acute inflammation of orbit |  | 376.0x | H05.0x |
| Infective otitis externa |  | 380.1x | H60.0x, H60.1x, H60.2x, H60.3x, , H62.4x, |
| Suppurative or unspecified otitis media |  | 382.xx | H66.xx |
| Acute mastoiditis |  | 383.0x | H70.0x |
| Acute nasopharyngitis (common cold) |  | 460.xx | J00.xx |
| Acute sinusitis |  | 461.xx | J01.xx |
| Acute pharyngitis |  | 462.xx | J02.xx |
| Acute tonsillitis |  | 463.xx | J03.xx |
| Acute laryngitis and tracheitis |  | 464.xx | J04.xx, J05.xx |
| Acute upper respiratory tract infection of multiple or unspecified sites |  | 465.xx | J06.xx |
| Acute bronchitis and bronchiolitis |  | 466.xx | J20.xx, J21.xx |
| Chronic sinusitis |  | 473.xx | J32.xx |
| Chronic disease of tonsils and adenoids |  | 474.xx | J35.xx |
| Chronic laryngitis and laryngotracheitis |  | 476.xx | J37.xx |
| Viral pneumonia |  | 480.xx | J12.xx |
| Pneumococcal pneumonia |  | 481.xx | J13.xx, J18.1x |
| Other bacterial pneumonia |  | 482.xx | J15x, J14x |
| Pneumonia due to other specified organism |  | 483.xx | J16.xx |
| Pneumonia in infectious diseases classified elsewhere |  | 484.xx | J17.xx |
| Bronchopneumonia organism unspecified |  | 485.xx | J18.0x |
| Pneumonia organism unspecified |  | 486.xx | J18.xx |

Table e2: ICD-9-CM and ICD-10-CA codes for identification of infection-related hospital admissions and physician visits (continued)

| **infection** |  | **ICD9** | **ICD10** |
| --- | --- | --- | --- |
| Influenza |  | 487.xx | J11.xx, J10.xx, J09.xx |
| Bronchitis |  | 490.xx | J40.xx |
| Infections of the kidney (pyelonephritis renal abscess) |  | 590.xx | N10.xx, N11. xx, N12.xx,, N15.1x, N28.84, N28.85, N28.86, N16.xx |
| Cystitis |  | 595.xx | N30.xx |
| Urethritis |  | 597.xx | N34.xx |
| Urinary tract infection, site not specified |  | 599.0x | N39.0x |
| Inflammatory disease of prostate (prostatitis) |  | 601.xx | N41.xx |
| Carbuncle and furuncle |  | 680.xx | L02.xx |
| Cellulitis and abscess of finger and toe |  | 681.xx | L03.xx |
| Other cellulitis and abscess |  | 682.xx | L03.xx |
| Acute lymphadenitis |  | 683.xx | L04.xx |
| Impetigo |  | 684.xx | L01.xx |
| Other local infections of skin and subcutaneous tissue |  | 686.xx | L08.xx, L88.xx, L98.0x, |
| Septic arthritis |  | 711.0x | M00.xx, M01.xx |
| Osteomyelitis periostitis and other infections involving bone |  | 730.xx | M86.xx, M46.2x  M46.3x, M89.6x |
| Bacteremia |  | 790.7x | [R78.81](http://www.icd10data.com/ICD10CM/Codes/R00-R99/R70-R79/R78-/R78.81) |
| Viremia |  | 790.8x | B34.9x |

Table e3. Drug identification numbers (DINs) for disease-modifying therapies available during the study period

| - **Disease-modifying therapy** | - **DIN** |
| --- | --- |
| - Betaseron | - 02169649 |
| - Extavia | - 02337819 |
| - Avonex | - 02237770, 02269201 |
| - Rebif | - 02281708, 02277492, 02237317, 02237319, 02237320, 02237320, 02318253, 02318261 |
| - Copaxone | - 02233014, 02245619 |
| - Tysabri | - 02286386 |
| - Gilenya | - 02365480 |
| - Tecfidera | - 02404508 |
| - Aubagio | - 02416328 |
| - Lemtrada | - 02418320 |
| - Plegridy | - 0244399 |

Table e4. Frequency (percent) of diagnostic indicators according to numbers of days between diagnostic codes

|  | **Days Apart** |  |  |
| --- | --- | --- | --- |
| **Diagnosis codes (≥2)** | **≥90** | **≥60** | **≥120** |
| Visual disturbance | 151 (8.6) | 166 (9.4) | 143 (8.1) |
| Blindness and low vision | 15 (0.85) | 23 (1.3) | 13 (0.74) |
| Abnormalities of gait and mobility | 0 (0) | 0 (0) | 0 (0) |
| Speech disturbances not elsewhere classified | 0 (0) | 0 (0) | 0 (0) |
| Limitation of activities due to disability | 0 (0) | 0 (0) | 0 (0) |
| Need for assistance due to reduced mobility | 0 (0) | 0 (0) | 0 (0) |
| Hemiplegia and hemiparesis | 12 (0.68) | 12 (0.68) | 11 (0.62) |
| Other paralytic syndromes/ Quadriplegia and quadriparesis | 166 (9.4) | 178 (10.1) | 153 (8.7) |
| Falls | 10 (0.57) | 11 (0.62) | 10 (0.57) |
| Dementia in other diseases classified elsewhere | 0 (0) | 0 (0) | 0 (0) |
| Incontinence – urinary or fecal | 12 (0.68) | 12 (0.68) | 12 (0.68) |
| Malaise and fatigue | 35 (2.0) | 42 (2.4) | 34 (1.9) |
| Pressure ulcer | 17 (0.96) | 19 (1.1) | 15 (0.85) |
| Spasticity | 26 (1.5) | 27 (1.5) | 23 (1.3) |
| Skin sensation disturbance | s | 7 (0.40) | s |
| Dysarthria | 0 (0) | 0 (0) | 0 (0) |
| Dysphagia | 0 (0) | 0 (0) | 0 (0) |

s = suppressed cell sizes <5 to protect privacy and confidentiality

Table e5. Performance of Disability Indicator for Severe Disability Measured Within 365 days after EDSS record, and within study window.

| **Indicator** | **Sens**  **(95%CI)** | **Spec**  **(95%CI)** | **PPV**  **(95%CI)** | **NPV**  **(95%CI)** | **Kappa**  **(95%CI)** |
| --- | --- | --- | --- | --- | --- |
| Visual disturbance | 7.0  (3.46, 10.5) | 91.8  (89.5, 94.1) | 24.1  (13.1, 35.1) | 72.7  (69.3, 76.0) | 0  (0, 0.04) |
| Other paralytic syndromes | 25.5  (19.5, 31.5) | 96.3  (94.7, 97.9) | 71.8  (61.4, 82.3) | 77.9  (74.7, 81.0) | 0.27  (0.20, 0.35) |
| Falls | 2.48  (0.33, 4.62) | 99.8  (99.5, 100) | 83.3  (53.5, 100) | 73.6  (70.4, 76.7) | 0.03  (0.00, 0.06) |

Table e6. Logistic regression-based algorithm for severe disability excluding individuals with treated relapses (regression coefficients shown)*

| **Model** | **Intercept** | **Age** | **Home Care** | **Long-term care** | **Rehabili-tation** | **Visual disturbance** | **Other paralytic syndromes** | **Spasticity** | **C-statistic** | **Optimism-corrected C-statistic** | **AIC** |
| --- | --- | --- | --- | --- | --- | --- | --- | --- | --- | --- | --- |
| A | -3.7979 | 0.0470 | 1.9268 | 1.7508 | 1.4598 | -1.2937 | 0.9418 | 2.2141 | 0.841 | 0.838 | 1193.7 |
| B | -3.8936 | 0.0476 | 1.9006 | 1.6688 | 1.424 |  | 0.9362 | 2.0889 | 0.836 | 0.834 | 1209.7 |
| C | -3.7864 | 0.0477 | 2.0706 | 1.7686 |  | -1.2431 | 1.0638 | 2.2581 | 0.833 | 0.831 | 1218.3 |

*Requires calculating probability of severe disability, where probability ≥0.25 indicates severe disability, AIC = Akaike’s Information Criterion

Table e7. Truncated regression-based algorithms for disability as a continuous variable excluding individuals with treated relapses

| **Model** | **Intercept** | **Age** | **Home Care** | **Long-term care** | **Rehabili-tation** | **Visual disturbance** | **Other paralytic syndromes** | **Spasticity** | **Mean Diff** | **MSE** | **Pseudo-R^2^** |
| --- | --- | --- | --- | --- | --- | --- | --- | --- | --- | --- | --- |
| A^a^ | 1.21442 | 0.040205 | 2.64715 | 1.42122 | 1.11397 | -0.43309 | 1.36400 | 2.01848 | -0.0683  (-0.1687, 0.0337) | 2.08 | 41.8% |
| B | 1.14223 | 0.04340 | 2.80757 | 1.40136 |  |  | 1.48683 | 2.12595 | -0.0686  (-0.1708, 0.0336) | 2.11 | 40.6% |
| C | 1.19834 | 0.04292 | 2.80578 | 1.42469 |  | -0.42320 | 1.48440 | 2.15350 | -0.0683  (-0.1703, 0.0337) | 2.10 | 40.8% |

1. Did not improve adding non-linear term for age, all p<0.0001. MSE = Mean standard error

Table e8. Annual rates of health care use during the study period due to any cause and due to infection

| **Fiscal year** | **All cause** | | | **Infection** | | |
| --- | --- | --- | --- | --- | --- | --- |
|  | **No. events** | **Person-years** | **Rate** | **No.**  **events** | **Person-years** | **Rate** |
| *Physician Visits* | | | | | | |
| 2014/15 | 14228 | 1663.46 | 8.55  (8.41, 8.69) | 844 | 1663.46 | 0.51  (0.47, 0.54) |
| 2015/16 | 14632 | 1715.96 | 8.37  (8.23, 8.51) | 787 | 1715.96 | 0.46  (0.43, 0.49) |
| 2016/17 | 14193 | 1757.50 | 8.08  (7.94, 8.21) | 776 | 1757.50 | 0.44  (0.41, 0.47) |
| *Hospitalizations* | | | | | | |
| 2014/15 | 230 | 1663.46 | 0.14  (0.12, 0.16) | 59 | 1663.46 | 0.035  (0.027, 0.046) |
| 2015/16 | 212 | 1715.96 | 0.12  (0.11, 0.14) | 64 | 1715.96 | 0.037  (0.029, 0.048) |
| 2016/17 | 222 | 1757.50 | 0.13  (0.11, 0.14) | 70 | 1757.50 | 0.040  (0.032, 0.050) |

Table e9. Association of Expanded Disability Status Scale scores and disability status algorithms with rates of hospitalization and physician visits

|  | **Hospitalizations** | | | | **Physician Visits** | | | | | |  |
| --- | --- | --- | --- | --- | --- | --- | --- | --- | --- | --- | --- |
|  | **Any cause** | | **Infection** | | **Any cause** | | | **Infection** | | |  |
|  | **Unadjusted**  **RR (95%CI)** | **Adjusted**  **RR (95%CI)** | | **Adjusted**  **RR (95%CI)** | **Unadjusted**  **RR (95%CI)** | **Adjusted**  **RR (95%CI)** | | | **Adjusted**  **RR (95%CI)** | |  |
| *EDSS (Continuous)* | 1.25  (1.20, 1.30) | 1.27  (1.20, 1.34) | | 2.04  (1.77, 2.36) | 1.03  (1.01, 1.05) | 1.02  (1.00, 1.03) | | | 0.97  (0.92, 1.02) | |  |
| *EDSS (categorized)* |  |  | |  |  |  | | |  | |  |
| 0-3.0 | - | - | | - | - | - | - | | |  |  |
| 3.5-5.5 | 1.23  (0.86, 1.77) | 1.28  (0.88, 1.85) | | 0.92  (0.25, 3.40) | 1.11  (1.01, 1.23) | 1.08  (0.98, 1.19) | | | 0.81  (0.61, 1.07) | | |
| 6.0-9.5 | 3.25  (2.56, 4.13) | 3.29  (2.44, 4.45) | | 18.24  (8.60, 38.71) | 1.12  (1.02, 1.22) | 1.01  (0.93, 1.10) | | | 0.82  (0.65, 1.04) | |  |
|  |  |  | |  |  |  | | |  | |  |
| *Disability-Cont (continuous)* | 1.31  (1.26, 1.36) | 1.34  (1.27, 1.42) | | 1.91  (1.74, 2.10) | 1.07  (1.05, 1.10) | 1.06  (1.04, 1.09) | | | 1.07  (0.99, 1.14) | |  |
| 0-3.0 | - | - | | - | - |  | | |  | |  |
| *Disability-Cont (categorized)* | | | | | | | | | | |  |
| 3.5-5.5 | 0.98  (0.76, 1.27) | 1.00  (0.71, 1.41) | | 2.70  (1.05, 6.91) | 1.01  (0.93, 1.09) | 0.93  (0.83, 1.04) | | | 0.82  (0.65, 1.04) | |  |
| 6.0-9.5 | 3.52  (2.75, 4.52) | 3.47  (2.37, 5.07) | | 38.39  (14.79, 99.68) | 1.31  (1.17, 1.46) | 1.11  (0.97, 1.28) | | | 0.91  (0.71, 1.15) | |  |
| Disability-SEV | 3.17  (2.61, 3.85) | 3.34  (2.57, 4.36) | | 17.8  (9.28, 34.2) | 1.22  (1.13, 1.32) | 1.10  (1.01, 1.20) | | | 1.17  (0.95, 1.44) | |  |

Figure e1. Bland-Altman plot for Disability-Cont Versus Clinical Expanded Disability Status Scale (EDSS) Score from Model A


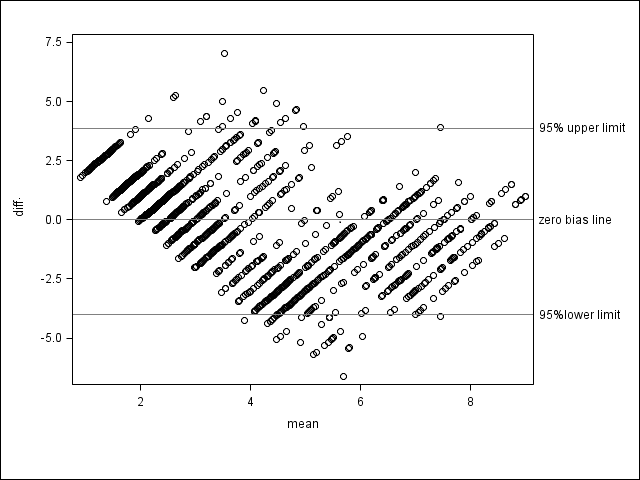


Diff = difference between Disability-Cont (model-predicted EDSS) and clinical EDSS, Mean = mean of Disability-Cont and clinical EDSS
